# Brain-Derived Neurotrophic Factor in Chronic Periodontitis

**DOI:** 10.1155/2014/373765

**Published:** 2014-12-21

**Authors:** Jôice Dias Corrêa, Daniele Sirineu Pereira, Mila Fernandes Moreira Madeira, Celso Martins Queiroz-Junior, Danielle Glória Souza, Mauro Martins Teixeira, José Eustáquio Costa, Antônio Lúcio Teixeira, Tarcília Aparecida da Silva

**Affiliations:** ^1^Department of Oral Surgery and Pathology, School of Dentistry, Federal University of Minas Gerais, Antonio Carlos Avenue 6627, 31.270-901 Belo Horizonte, MG, Brazil; ^2^Department of Physical Therapy, School of Physical Education, Physical Therapy and Occupational Therapy, Federal University of Minas Gerais, Antonio Carlos Avenue 6627, 31.270-901 Belo Horizonte, MG, Brazil; ^3^Department of Microbiology, Biological Sciences Institute, Federal University of Minas Gerais, Antonio Carlos Avenue 6627, 31.270-901 Belo Horizonte, MG, Brazil; ^4^Department of Biochemistry and Immunology, Biological Sciences Institute, Federal University of Minas Gerais, Antonio Carlos Avenue 6627, 31.270-901 Belo Horizonte, MG, Brazil; ^5^Department of Clinical Medicine, Faculty of Medicine, Federal University of Minas Gerais, Professor Alfredo Balena Avenue 110, 30130-100 Belo Horizonte, MG, Brazil

## Abstract

Brain-derived neurotrophic factor (BDNF) is a member of the neurotrophic factor family. Outside the nervous system, BDNF has been shown to be expressed in various nonneural tissues, such as periodontal ligament, dental pulp, and odontoblasts. Although a role for BDNF in periodontal regeneration has been suggested, a function for BDNF in periodontal disease has not yet been studied. The aim of this study was to analyze the BDNF levels in periodontal tissues of patients with chronic periodontitis (CP) and periodontally healthy controls (HC). All subjects were genotyped for the rs4923463 and rs6265 *BDNF* polymorphisms. Periodontal tissues were collected for ELISA, myeloperoxidase (MPO), and microscopic analysis from 28 CP patients and 29 HC subjects. BDNF levels were increased in CP patients compared to HC subjects. A negative correlation was observed when analyzing concentration of BDNF and IL-10 in inflamed periodontium. No differences in frequencies of BDNF genotypes between CP and HC subjects were observed. However, *BDNF* genotype GG was associated with increased levels of BDNF, TNF-*α*, and CXCL10 in CP patients. In conclusion, BDNF seems to be associated with periodontal disease process, but the specific role of BDNF still needs to be clarified.

## 1. Introduction

Periodontal health can be described as “a dynamic state where the activity of proinflammatory/antimicrobial cytokines to control infection is optimally balanced by anti-inflammatory mechanisms to prevent unwarranted inflammation” [1]. In subjects susceptible to periodontal disease (PD), an imbalance of the inflammatory response results in excessive production of proinflammatory cytokines and the subsequent loss of periodontal attachment [2]. On the other hand, controlling mechanisms prevent excessive tissue damage [3]. Furthermore, the release of tissue regenerating factors may contribute to periodontal regeneration by regulating the function of periodontal ligament cells, endothelial cells, and cementoblasts [4]. In this setting, neurotrophin brain-derived neurotrophic factor (BDNF) has been reported to enhance periodontal tissue regeneration [4, 5]. BDNF is a member of the neurotrophin family which is expressed by vascular endothelium and osteoblastic, immune, and neuronal cells [6]. BDNF is reported to be involved in the joint inflammatory process and its production is increased in response to proinflammatory cytokines [6].

Although a role for BDNF in periodontal regeneration has been proposed, no information is available concerning BDNF and periodontal disease. The aim of this study was to measure the levels of BDNF in periodontal tissues from patients with chronic periodontitis. The presence of polymorphisms rs6265 and rs4923463 of the* BDNF* gene and its correlation with inflammatory and clinical parameters were also assessed.

## 2. Material and Methods

### 2.1. Subjects and Sample Collection

Twenty-eight patients with CP, treated at the Periodontal Clinic, School of Dentistry, at Universidade Federal de Minas Gerais (UFMG, Brazil), were enrolled in this study. Periodontal tissue samples were obtained during periodontal surgery. Patients in this study met the following inclusion criteria: previous history of CP, diagnosed according to previously described criteria: (1) exhibiting more than one tooth with probing depth higher than 5 mm, (2) exhibiting more than two sites with clinical attachment loss deeper than 6 mm, and (3) exhibiting lesions distributed in more than two teeth in each quadrant [7]. Patients who met the following criteria were excluded: (1) having a history of smoking, (2) use of antibiotic, (3) usage of anti-inflammatory and/or immunosuppressive medications during the 6 preceding months, and (4) a history of any systemic diseases (i.e., immunologic and autoimmune disorders, diabetes mellitus). The control group (HC) comprised 29 age and gender matched periodontally healthy patients enrolled for third molar removal surgery.

Periodontal examination was performed in both groups of patients, CP and HC, at the initial visit to determine probing depth (PD), clinical attachment loss (CAL), and bleeding on probing (BOP). The BOP was considered positive if bleeding occurred within 30 seconds after probing [8]. Measurements were performed full-mouth at 6 sites per tooth (mesiobuccal, midbuccal, distobuccal, mesiolingual, midlingual, and distolingual). All the measurements were performed by the same examiner. At the time of the examination a peripheral blood sample was taken from each patient and processed for polymorphism determination.

Written informed consent was obtained from all patients. The study protocol was approved by the Institutional Ethics Committee (Protocol 324/08).

### 2.2. Inflammatory Infiltrate Evaluation

Periodontal tissue samples from periodontal pockets or healthy oral mucosa extracted during surgery of impacted third molars were fixed in 10% buffered formalin, embedded in paraffin wax, and cut longitudinally (3 *μ*m). The sections were deparaffinized, rehydrated, and stained with H&E for evaluation of the inflammatory infiltrate. Inflammatory cells were counted in four fields in two independent sections, using a light microscope (Axioskop 40 ZEISS; Carl Zeiss, Gottingen, Germany) at 400x magnification. Data were expressed as total inflammatory cells/field.

### 2.3. ELISA

The concentrations of the IL-17A, BDNF, IL-10, and TNF-*α* and the chemokine CXCL10 were measured in periodontal tissues by enzyme-linked immunosorbent assay (ELISA) using commercially available kits (R&D Systems, Minneapolis, MN, USA). The assay was performed according to manufacturer's instructions. The wavelength of the microplate reader was 492 nm. The lower limit of detection for each cytokine was 15 pg/mL, 3.9 pg/mL, 5.5 pg/mL, 20 pg/mL, and 4.5 pg/mL, respectively, for IL-17A, IL-10, TNF-*α*, BDNF, and CXCL10. The data were determined using a standard curve prepared for each assay and expressed as picograms of cytokine/chemokine per 100 mg of tissue.

### 2.4. Myeloperoxidase

Periodontal tissue samples were also used for determination of myeloperoxidase (MPO) activity, a neutrophil enzyme marker, as described earlier [9]. The MPO activity in homogenized periodontal tissues was evaluated by enzymatic reaction and absorbance was measured at 450 nm. The MPO content was expressed as relative units calculated from standard curves based on MPO activities from 5% casein peritoneal-induced neutrophils assayed in parallel.

### 2.5. DNA Isolation and Genotyping Analysis

Total genomic DNA was extracted from blood samples using QIAamp DNA Blood Mini Kit (QIAGEN, Valencia, CA, USA) according to manufacturer's instructions. Quality, integrity, and quantity of DNA were analyzed by NanoDrop spectrophotometer (Thermo Scientific, Wilmington, DE, USA).

TaqMan genotyping assays were obtained from Applied Biosystems, Inc. (Foster City, CA, USA). The assay identification code for each respective SNP is* BDNF* rs6265 and rs4923463. All amplifications were carried out in an ABI 7900H thermal cycler (Applied Biosystems, Foster City, CA, USA) using TaqMan Genotyping Master Mix and following manufacturer's recommended amplification conditions.

### 2.6. Data Analysis

Data were expressed as mean ± standard deviation (SD). Chi-square test analysis was used to test for deviation of genotype frequencies from Hardy-Weinberg equilibrium. The levels of cytokines in periodontal tissues and the frequency of gene polymorphisms were compared by the Student's *t*-test and chi-square tests. *P* values < 0.05 were considered statistically significant. All data were analyzed using SPSS 17 (SPSS Inc., Chicago, IL, USA).

## 3. Results

### 3.1. Clinical and Inflammatory Parameters

**Figure 1:**
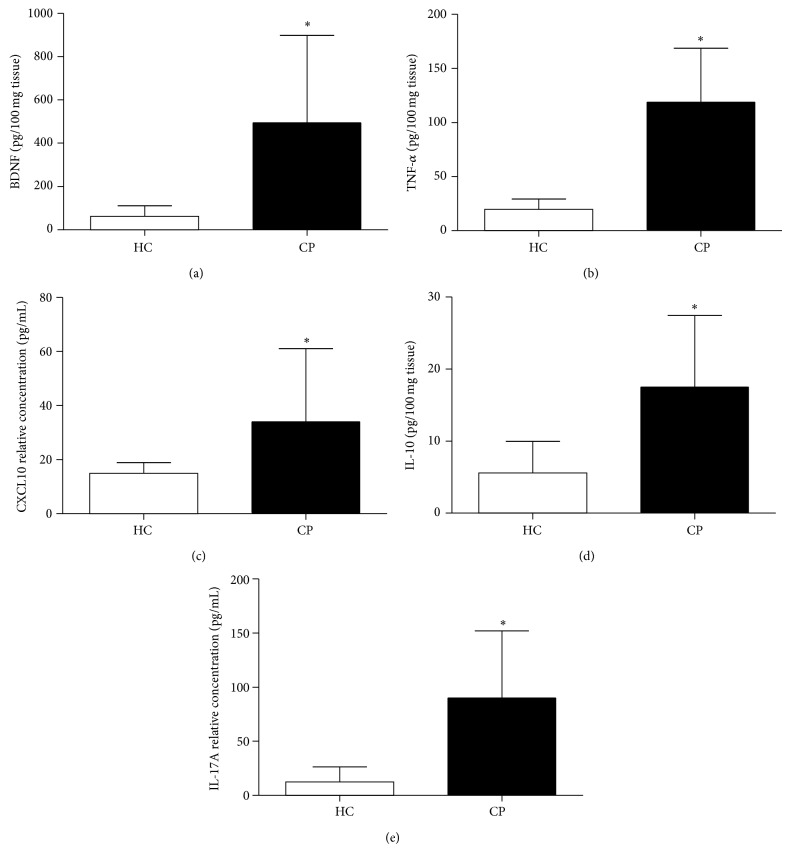
Levels of (a) BDNF, (b) TNF-*α*, (c) CXCL10, (d) IL-10, and (e) IL17A in the periodontal tissues from CP and HC subjects. ^*^Statistically significant difference at *P* < 0.05 in relation to HC (Student's *t*-test). HC: healthy control; CP: chronic periodontitis.

**Figure 2:**
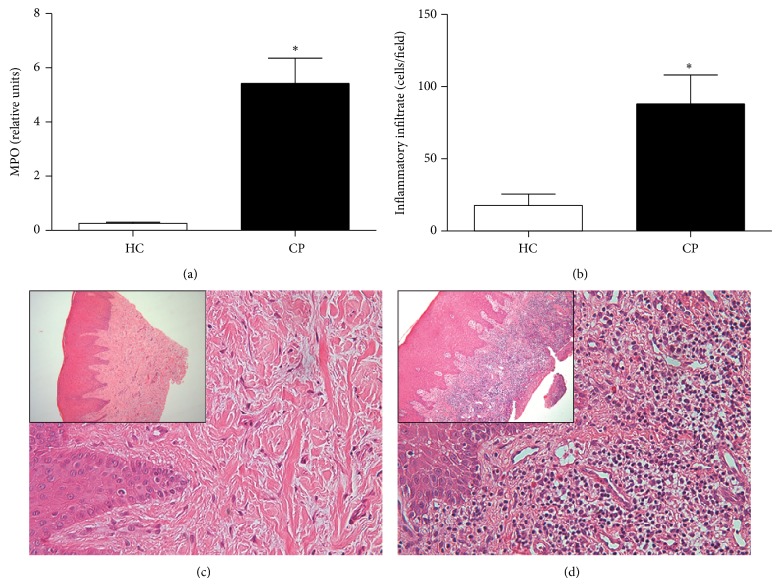
MPO activity (a). Number of inflammatory cells in the periodontal tissues from CP and HC subjects (b). Representative images of periodontal tissues from HC (c) and CP (d). H&E staining. Magnification: 400x. Insert magnification: 100x. ^*^Statistically significant difference at *P* < 0.05 in relation to HC (Mann-Whitney *U* test). HC: healthy control; CP: chronic periodontitis; MPO: myeloperoxidase.

The sample included in the current study was composed by age and gender matched groups. The clinical features PD, CAL, and BOP were significantly higher in the CP than in the HC group (*P* < 0.0001) (Table 1). The levels of IL-17A, CXCL10, IL-10, TNF-*α*, and BDNF in periodontal tissues were greater in CP patients than in controls (Figure 1). Moreover, the MPO activity and the inflammatory infiltrate in the periodontal tissues, characterized by polymorphonuclear and mononuclear leukocytes, were significantly higher in the CP than in the HC group (Figure 2).

The BDNF and IL-10 levels in periodontal tissues were negatively correlated (*R* = −0.691, *P* = 0.002), whereas no correlation between BDNF and IL-17A, TNF-*α*, CXCL10, or clinical parameters was observed (PD, CAL, and BOP).

### 3.2. Association among Polymorphisms and Clinical Periodontal Parameters

Following the clinical investigation, the frequencies of polymorphisms (*BDNF*) were assessed in blood samples of HC and CP subjects (Table 2). The frequency of these genotypes agreed with the Hardy-Weinberg equilibrium (*P* > 0.05). The distribution of the* BDNF* polymorphisms was similar between the groups (Table 2).

We also investigated whether some of these polymorphisms were associated with worse clinical periodontal parameters. As shown in Table 3, no differences in clinical parameters were found when comparing the genotypes.

### 3.3. *BDNF* Genotypes Show Differences in the Expression of BDNF and Inflammatory Mediators in Periodontal Tissues

**Figure 3:**
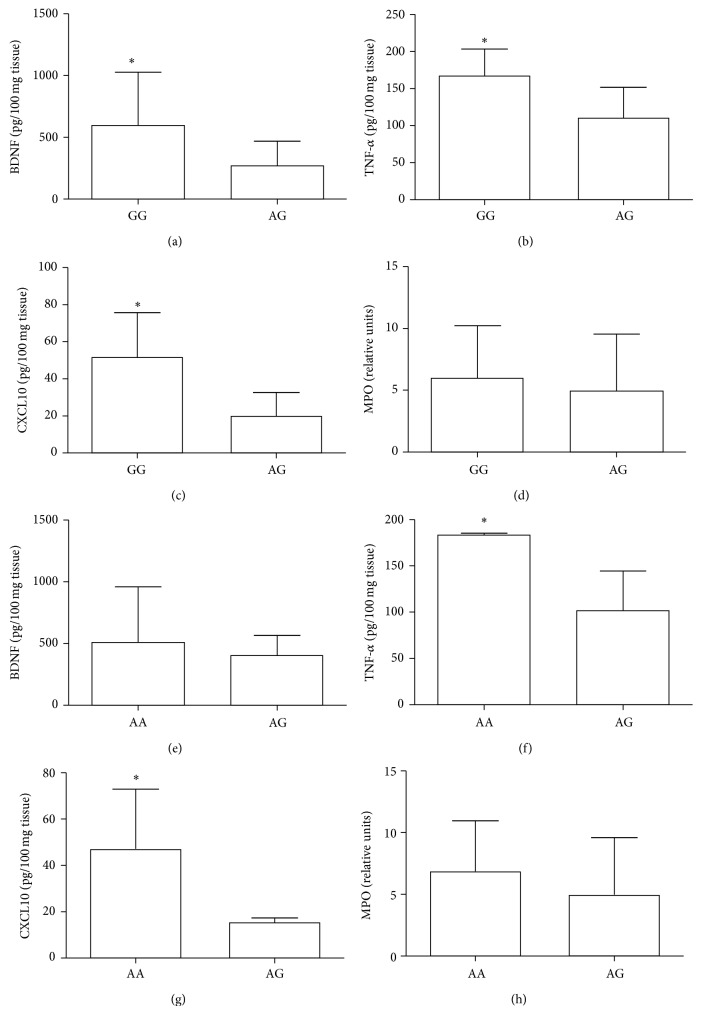
Levels of BDNF and inflammatory mediators in CP patients according to each* BDNF* genotype. (a) Levels of BDNF, (b) TNF-*α*, and (c) CXCL10 and (d) MPO in periodontal tissues samples with* BDNF* rs6265 polymorphism. (e) Levels of BDNF, (f) TNF-*α*, and (g) CXCL10 and (h) MPO in periodontal tissues samples with* BDNF* rs4923463 polymorphism. ^*^Statistically significant difference at *P* < 0.05 in comparison to the AG genotype (Student's *t*-test).

The levels of BDNF and the inflammatory mediators CXCL10 and TNF-*α* were increased in GG genotype of* BDNF* rs6265 polymorphism (Figures 3(a), 3(b), and 3(c)), but MPO levels did not alter significantly (Figure 3(d)). In* BDNF* rs4923463 polymorphism the levels of BDNF and MPO did not differ, but the levels of CXCL10 and TNF-*α* were higher in patients with AA genotype (Figures 3(e)–3(h)).

## 4. Discussion

A wide range of nonneural cells in peripheral tissues or in the immune system expresses neurotrophins and their receptors. Thus, the mitogenic and immune regulatory functions of neurotrophins have been discussed [6, 10–12]. The neurotrophin BDNF is reported to be involved in inflammatory reactions [13], and its production is increased in response to proinflammatory cytokines [14]. The present study is the first to demonstrate that BDNF levels were increased in periodontal tissues from chronic periodontitis compared to healthy subjects. In agreement with our findings, BDNF was found in high levels in the plasma of patients with osteoarthritis [15] and in patients with rheumatoid arthritis [6]. While some authors [15] reported that BDNF levels were significantly correlated with self-reported pain, others [6] did not find association between BDNF and clinical parameters of arthritis. We did not observe association of BDNF levels with clinical parameters.

Several studies analyzed BDNF rs6265 polymorphisms in psychiatric disorders [16–18]. To date, few studies examined the rs4923463 polymorphism [16, 18–20]. The rs4923463 is adjacent to the functional polymorphism rs6265 Val66Met [19]. While one study found correlation between SNP rs4923463 and attention-deficit/hyperactivity disorder, schizophrenia, and risk of suicide in bipolar disorder, another study did not find any correlation between schizophrenia and rs4923463 polymorphism [18].

Previously, two studies have evaluated the effects of BDNF polymorphisms in bone [21, 22], but there are no available studies in periodontal disease. In the present study we did not find differences in* BDNF* genotype distribution between patients with CP and controls. Nevertheless, we found that subjects with GG (rs6265) genotype expressed higher levels of BDNF in periodontal tissues, in agreement with a previous report showing that BDNF-M66 variant alters intracellular trafficking and impairs BDNF secretion [23]. On the other hand, rs6265 polymorphism was not associated with production of BDNF by immune cells from multiple sclerosis patients [24]. Interestingly, the SNP rs6265 was reported as a phosSNP, which means this SNP regulates protein phosphorylation [22]. The SNP rs6265 affects substrate-kinase interaction between BDNF protein and CHEK2 kinase and regulates BDNF phosphorylation at site T62. Subjects AA genotype carriers exhibited lower bone mineral density compared to G carriers [22]. Specifically,* BDNF*-*V66* (major allele* G* at rs6265) transfection significantly increases expression of osteoblast specific markers (*OPN*,* BMP2*, and* ALP*) and promotes osteoblast differentiation and maturation in cell culture [22]. An association of rs6265 with bone metabolism was also suggested in the largest meta-analysis involving 32,961 individuals of European and East Asian ancestry. The study showed that rs6265 was associated with femoral neck bone mineral density. They found that homozygous minor allele A carriers (AA) have significantly decreased BMD compared to major allele G carriers (GA and GG) [21].

It has been reported that BDNF is able to induce an increase in IL-10 expression [25]. However, a negative correlation between the production of BDNF and IL-10 was observed in samples from patients with periodontitis. IL-10 can inhibit the release of proinflammatory cytokines from monocytes/macrophages and can therefore inhibit the lipopolysaccharide- and IFN-*γ*-induced secretion of inflammatory cytokines (e.g., TNF-*α*, IL-1*β*, IL-6, CXCL8, and others) [26]. In periodontal disease, IL-10 is thought to be associated with lower disease severity [27].

Previous studies demonstrated that BDNF induces periodontal tissue regeneration by activation of cementoblasts differentiation, vascular endothelial cell migration [4], and also has a positive effect on bone remodeling [5, 28]. This data together suggested that BDNF has a role in bone remodeling and any change in this neurotrophin levels could have an impact in bone repair.

Finally, we observe that CP subjects with GG (rs6265) and AA (rs4923463) genotypes demonstrated increased levels of TNF-*α* and CXCL10. CXCL10 has several roles, such as chemoattraction of macrophages, T cells, NK cells, and dendritic cells [2]. Some of these cells are important for tissue repair [29]. In addition, previous studies showed that exposure to BDNF substantially and synergistically enhanced TNF-*α* levels* in vitro* [30], and TNF-*α* preconditioning increased proliferation, mobilization, and osteogenic differentiation* in vitro* [31]. We can hypothesize that the concomitant increase of BDNF, TNF-*α*, and CXCL10 in patients with the GG genotype may be an attempt of the host to induce periodontal healing. So, maybe if these patients were examined after periodontal treatment, they could display higher and better levels of tissue regeneration compared to patient who do not exhibit the GG genotype.

In conclusion, BDNF seems to be related to periodontal pathogenesis and also involved in tissue repair. The results obtained here provide a benchmark for future studies with a large cohort of patients to help strengthen and understand the influence of neurotrophins in periodontal disease.

## Figures and Tables

**Table 1:** Demographic and clinical features of the studied subjects.

	HC (*N* = 29)	CP (*N* = 28)	*P* value
Age (SD)	41.21 (8.4)	44.4 (8.5)	0.25
Gender (% F)	56.5	51.20	0.63
PD (SD)	2.60 (0.7)	4.46 (0.9)^*^	<0.0001
CAL (SD)	2.06 (0.9)	5.07 (0.8)^*^	<0.0001
BOP (SD)	5.7 (0.6)	31.6 (2.2)^*^	<0.0001

HC: healthy controls; CP: chronic periodontitis; SD: standard deviation; PD: probing depth; CAL: clinical attachment loss; BOP: bleeding on probing (% of sites).

^*^Significantly higher than control (*P* < 0.05, *χ* ^2^ test or Student's *t*-test).

**Table 2:** *BDNF* genotypes polymorphisms in patients with chronic periodontitis (CP) and healthy controls (HC).

Genotype	HC (%)	CP (%)	*P* value	OR (95% CI)
*BDNF* rs6265				
GG	88.88	83.33	*0.15 *	0.30 (0.05–1.67)
AG	11.12	16.67	*0.21 *	
*BDNF* rs4923463				
AA	88.88	76.00	*0.44 *	0.50 (0.08–3.01)
AG	11.12	24.00	*0.36 *	

OR: odds ratio; CI: confidence interval.

**Table 3:** Association between *BDNF* polymorphisms and clinicopathological features of chronic periodontitis.

Genotype	PD (mm)	*P* value	CAL (mm)	*P* value	BOP (%)	*P* value
*BDNF* rs6265						
GG	4.58	*0.586 *	5.42	*0.744 *	30.80	*0.115 *
AG	4.35		5.35		22.46	
*BDNF* rs4923463						
AA	4.46	*0.729 *	5.21	*0.734 *	30.81	*0.323 *
AG	4.60		5.30		25.37	
